# Gender-specific associations between slow wave sleep and cardiovascular risk factors: too early to tell?

**DOI:** 10.1093/sleep/zsae264

**Published:** 2024-11-13

**Authors:** Sogol Javaheri, Susan Redline

**Affiliations:** Brigham and Women’s Hospital, Division of Sleep Medicine, Boston MA, USA; Brigham and Women’s Hospital, Division of Sleep Medicine, Boston MA, USA

The importance of sleep to cardiovascular health has been increasingly recognized, and sex and gender differences in sleep and cardiovascular disorders have also garnered increasing attention. Biological sex and gender identity influence biological and psycho-social risk factors for both sleep disorders and cardiovascular disease [1]. However, the causal associations linking sex/gender-associated risk factors with sleep disorders and cardiovascular disease are poorly understood [2, 3].

In an article published in this issue of *SLEEP*, Taporoski et al. cross-sectionally evaluated sex-specific associations between sleep stages and cardiovascular risk factors (blood pressure and lipid levels) among 1102 Brazilian adults, mean age approximately 47 year (range 18–91) among women and men [4]. They did not find any sex-specific differences in sleep stage and lipid levels but found an association between stage N3 and elevated blood pressure in women but not men. While this article highlighted the importance of considering sex differences in sleep and their associations with cardiovascular risk factors—and the possibility that features of inadequate sleep may better predict cardiovascular disease risk in females compared to males—it is important not to conclude that reduced N3 sleep only adversely impacts females. Prior literature has demonstrated that reduced N3 sleep is associated with incident hypertension in both females and males using data from the Sleep Heart Health Study (SHHS) [5] as well as in the cohort of males from the Outcomes of Sleep Disorders in Older Men (MrOS) study [6].

In the MrOS cohort, percentage N3 sleep was modeled by quartiles and was inversely associated with odds of incident HTN in 784 community-dwelling men ≥ 65 years of age after adjustment for age, race, study site, body mass index, sleep duration, sleep fragmentation, and indices of sleep-disordered breathing [6]. In the SHHS cohort (*n* = 1850), N3 sleep was also categorized into quartiles to account for potential nonlinear associations, and logistic regression models were adjusted for age, sex, race, site, waist circumference, alcohol use, and pack years of smoking. Despite modeling different thresholds, there was no evidence for sex difference in the association between stage N3 sleep and blood pressure in the SHHS cohort [5].

The findings from the SHHS and MrOS studies highlight the need to consider potential non-linear relationships in the associations between sleep metrics and cardiovascular risk factors. Since males and females often have different statistical distributions of physiological traits, there is also a need to utilize a sex-specific approach to define thresholds for normality. For example, in the data presented by Taporoski et al. [4], the distributions of N3 sleep differed by sex: (males: mean 36.7, standard deviation 33.8; women: mean 53.1, and standard deviation 36.8). Using only linear modeling and failing to consider differences in the underlying distributions by sex may have masked a potential relationship between N3 and cardiovascular risk markers in men in the Brazilian cohort.

As the authors recognized, insomnia is more common in women than men, and has also been associated with HTN [4]. It would be important to know if the reductions in N3 sleep were driven by data from the women with insomnia or self-reported poor sleep quality. In other words, does the association between N3 and hypertension also reflect perceived poor sleep quality characteristic of insomnia, or is the association of N3 sleep with cardiovascular risk similar for women regardless of symptoms? Sleep is multi-dimensional, and it is critical to consider both perceived and device-based assessments of sleep.

Additionally, Taporoski et al. [4] did not explicitly consider the influence of sex versus gender on their study outcomes, nor did they control for alcohol use and smoking, two measures that can affect N3 sleep, operating as confounders and/or effect moderators in the associations between sleep and cardiovascular risk markers [7]. Failure to consider these two lifestyle and health behaviors may also obscure sex/gender differences. Additional consideration of the “gendered environment”- psycho-social and other factors that differ in men and women- is also needed to fully understand the complex associations between sleep and cardiovascular disease and identify potential modifiable targets to improve sleep health in diverse samples. The sample in Taporoski included individuals across a wide age span. Both sleep and sex/gender effects vary across the life span [2], and it is critical that a life course approach be applied in future research. While obstructive sleep apnea (OSA) was adjusted for with use of a binary AHI variable (AHI > 15), OSA may be underestimated in women due to the shorter events that occur in women and a greater propensity for REM-dominant OSA [8]. Use of sex/gender appropriate OSA metrics may better adjust for confounding influences of OSA in both males and females.

Finally, we are increasingly understanding the limitations of summary metrics of sleep stages derived from manual scoring. Quantitative EEG metrics are able to identify pathological slowing of sleep and slow wave oscillations, and better quantify delta sleep and its temporal dynamics. These increasingly powerful EEG tools provide a window into complex neurophysiology including control of the autonomic nervous system, and there is evidence that men and women differ in autonomic and immune processing relevant for cardiovascular disease. Using quantitative EEG metrics may better help understand physiologic markers of cardiovascular health outcomes and sex differences [9].

This article highlights the need to carefully consider both sex and gender as factors that influence and may moderate sleep as a cardiovascular disease risk factor. The National Institutes of Health and its Office of Women’s Health Research are actively catalyzing research that addresses sex and gender differences, noting that the “time is now” to advance the knowledge of both the biological and the multiple psycho-social factors associated with sex and gender that drive disease and moderate treatment effects. Now indeed is the time for careful consideration of sex and gender differences in sleep health and their influence on cardiovascular disease and other health outcomes.
